# Sex Differences in Sleep Architecture After Traumatic Brain Injury: Potential Implications on Short-Term Episodic Memory and Recovery

**DOI:** 10.1089/neur.2023.0093

**Published:** 2024-01-03

**Authors:** Stefanie N. Howell, Grace S. Griesbach

**Affiliations:** ^1^ Centre for Neuro Skills, Bakersfield, California, USA.; ^2^ Department of Neurosurgery, David Geffen School of Medicine at the University of California, Los Angeles, California, USA.

**Keywords:** melatonin, memory, REM, sex differences, sleep, TBI

## Abstract

Sleep-wake disturbances (SWDs) are common after TBI and often extend into the chronic phase of recovery. Such disturbances in sleep can lead to deficits in executive functioning, attention, and memory consolidation, which may ultimately impact the recovery process. We examined whether SWDs post-TBI were associated with morbidity during the post-acute period. Particular attention was placed on the impact of sleep architecture on learning and memory. Because women are more likely to report SWDs, we examined sex as a biological variable. We also examined subjective quality of life, depression, and disability levels. Data were retrospectively analyzed for 57 TBI patients who underwent an overnight polysomnography. Medical records were reviewed to determine cognitive and functional status during the period of the sleep evaluation. Consideration was given to medications, owing to the fact that a high number of these are likely to have secondary influences on sleep characteristics. Women showed higher levels of disability and reported more depression and lower quality of life. A sex-dependent disruption in sleep architecture was observed, with women having lower percent time in REM sleep. An association between percent time in REM and better episodic memory scores was found. Melatonin utilization had a positive impact on REM duration. Improvements in understanding the impact of sleep-wake disturbances on post-TBI outcome will aid in defining targeted interventions for this population. Findings from this study support the hypothesis that decreases in REM sleep may contribute to chronic disability and underlie the importance of considering sex differences when addressing sleep.

## Introduction

Long-lasting memory impairments and emotional/behavioral issues that have an impact on life quality are prevalent after traumatic brain injury (TBI),^1–3^ with women reporting a higher percentage of symptoms.^4–6^ The cognitive and emotional sequelae of TBI may be additionally exacerbated by sleep-wake disturbances (SWDs).^7–9^ Prevalence of SWD is higher after a TBI compared to the general population.^8,10^ Sleep is known to be an essential component of daily living, impacting cognition and emotional regulation.^11–16^ Of interest is the role that sleep architecture plays in memory.^17^ Persons with no SWDs cycle through sleep stages that include wakefulness, non-rapid eye movement (NREM) sleep, and rapid eye movement (REM) sleep. NREM sleep is further divided into three stages (N1, N2, and N3) and includes oscillations associated with learning and memory.^16,18,19^ REM sleep has also been demonstrated to facilitate memory.^20–23^

Women are more likely to report insomnia and excessive daytime sleepiness.^24–28^ Moreover, sex differences in network organization during REM sleep have been observed in polysomnography (PSG) studies.^29,30^ In this observational study, we aimed to identify SWDs during the post-acute TBI period while considering sex as a biological variable. We hypothesized that disruptions in N3 and REM sleep stages would be associated with cognitive deficits. We also examined subjective quality of life, depression, and disability levels. Consideration was given to medications, given that a high number of these are likely to have secondary influences on sleep characteristics.

## Methods

### Study population

Study methods were approved by an institutional review board. We conducted a retrospective chart review of TBI patients having underwent a type I, fully attended overnight PSG while being treated at a post-acute rehabilitation facility (Centre for Neuro Skills, Dallas, TX; Bakersfield, CA). Exclusion criteria were: 1) under the age of 18 years or over the age of 60 years, 2) past diagnosis of neurodegenerative disease, 3) diagnosis of SWD before TBI, and 4) poor signal quality during PSG recording.

Medical records from the rehabilitation facility were reviewed to determine functional status during the period of the PSG sleep evaluation. The following assessments were utilized: Mayo Portland Adaptability Inventory 4 (MPAI); California Verbal Learning Test 2 (CVLT); Neurological Quality of Life Questionnaire (Neuro QoL) subscales; and the Beck Depression Inventory II (BDI). Medical records of medications taken during the night of the PSG that may impact sleep were documented for analysis.^31–37^

### Polysomnography

Sleep studies were performed by a Registered Polysomnographic Technologist (PSG-1100; Nihon Khoden, Irvine, CA). Recordings included frontal, central, and occipital electroencephalogram, electrooculogram, submentalis and anterior tibialis electromyography, body position, nasal and oral airflow, electrocardiogram, and oxygen saturation. Sleep staging and scoring was done according to the American Academy of Sleep Medicine manual, version 2.5. Apnea was scored at ≥3% oxygen desaturation. The Epworth Sleepiness Scale was utilized to detect subjective sleepiness.

## Statistical analysis

Statistical analyses were performed using PRISM software (version 9.5.1; GraphPad Software, San Diego, CA). Variables were evaluated for normality/lognormality. Two group comparisons were analyzed either through Mann-Whitney U or *t*-tests. All statistical tests were two-tailed. Data are reported as mean ± standard error of the mean (SEM). Mixed-model analysis was utilized to evaluate sex differences in Neuro QoL measures. For the analysis of sleep stages and memory, men and women were pooled to decrease the risk of type II errors attributable to low statistical power. Analyses of variance and corrected multiple comparisons were performed to compare REM sleep time below and above the normative percentage according to age.^38–41^ The following groups were compared: Low-Range-REM (<14% REM); Mid-Range-REM (14–22% REM); and High-Range-REM (>22% REM). Linear regression analysis was also utilized to determine the influence of REM on learning and memory.

Analysis of medication use during the sleep evaluation was performed in a binary manner. Fisher's exact tests were utilized to compare group differences in recorded medications. Power analysis indicated that sample sizes were insufficient to analyze the impact of REM sleep on memory while considering sex. To increase the sensitivity of detecting the potential effect of medications on sleep, men and women were pooled. Logistic regression was utilized to evaluate the association and probabilities between binary (medication utilization) and non-binary (percentage of time spent in REM sleep) variables. Significance was determined by a likelihood ratio test. Sensitivity and specificity for significant values were indicated with receiver operating characteristic (ROC) curves.

## Results

A total of 64 records were reviewed. Of those, 57 subjects met the inclusion criteria and consisted of 40 men and 17 women (percentage of 70% male). This study population is representative of the incidence of TBI among men. TBI rates in men are higher according to surveillance reports.^42^ Mean age in years at the time of the PSG was 41 ± 2 SEM with a median of 42 years. TBI chronicity in days at the time of PSG was 889 ± 300 SEM with a median of 141 days. Glasgow Coma Scale (GCS) was only recorded on 40% of subjects (mean GCS = 8 ± 1 SEM). To determine initial injury severity, additional injury information was obtained from the treating hospital, emergency room, and imaging reports. Only 7% of subjects did not require hospitalization post-TBI, suggesting that the remaining injuries were moderate to severe (Table 1). There were no significant sex differences in age or initial injury severity indicators. Most patients were taking a medication that could potentially have an impact on REM sleep and overall sleep quality (Table 2). No sex differences were observed in medication use.

**Table 1.** Demographics

Demographics	Men	Women	Overall
Age at PSG	43 ± 2	41 ± 2	41 ± 2
Skull fracture	24%	19%	22%
Isolated to head	41%	50%	50%
ER only, not admitted to the hospital	5%	12%	7%
Non-ICU hospitalization	23%	35%	25%
ICU hospitalization	72%	53%	68%

Data are shown as percentage or mean ± standard error of the mean.

PSG, polysomnography; ER, emergency room; ICU, intensive care unit.

**Table 2.** Medications During the Sleep Evaluation

	Men	Women
Antidepressants		
SSRI (citalopram, escitalopram, vilazodone, fluoxetine)	50%	35%
Cyclic (amitriptyline, mirtazapine)	5%	6%
SARI (trazodone)	23%	18%
Sedative-hypnotics		
Benzodiazepines (alprazolam, lorazepam, clonazepam)	18%	18%
Non-benzodiazepines (zolpidem, zaleplon, buspirone)	2.5%	12%
Melatonin	28%	24%
Antiepileptics		
First generation (Na^+^ valproate, phenytoin, carbamazepine, clonazepam)	25%	12%
Second generation (gabapentin, levetiracetam, lamotrigine, topiramate, oxcarbazepine)	38%	47%
Third generation (eslicarbazepine acetate, lacosamide, clobazam)	5%	0
Cardiovascular drugs		
Beta blockers (carvedilol, metoprolol, labetalol, propranolol)	18%	35%
Antihypertensives (losartan, clonidine, amlodipine, hydralazine, lisinopril, diltiazem, prazosin)	25%	41%
Amiodarone	0	6%
Antihistamines (diphenhydramine, loratadine, cetirizine)	10%	12%

Data are shown as percentage of subjects taking the indicating medication.

SSRI, selective serotonin reuptake inhibitor; SARI, serotonin antagonist and reuptake inhibitor.

### Functional sex differences during post-acute recovery period

Analysis of medical records revealed significant sex effects in levels of disability, depression, and quality of life during the PSG period. No sex effects were observed for CVLT scores. BDI values in women were indicative of moderate depression. Women also showed more disability, according to the MPAI, particularly in the Ability subscale (Table 3).

**Table 3.** Functional Sex Differences

	Men	Women
BDI^*^	**13 ± 1.5**	**22 ± 3.6**
MPAI Ability^*^	**55 ± 1.7**	**60 ± 2.0**
MPAI Adjustment	49 ± 1.3	54 ± 3.1
MPAI Participation	51 ± 1.8	56 ± 3.1
MPAI Total^**^	**52 ± 1.7**	**61 ± 2.4**

BDI scores (ranging from 0 to 63) and MPAI T scores are shown as mean ± standard error of the mean. Significance is demonstrated by ^*^p < 0.05, ^**^p < 0.005.

BDI, Beck Depression Inventory II; MPAI, Mayo Portland Adaptability Inventory.

Women reported a less desirable quality of life when analyzing scales where a high score indicated better self-reported health (*F*_(1,43)_ = 20.08, *p* < 0.0005), as well as those scales where a low score indicated better self-reported health (*F*_(1,43)_ = 7.88, *p* < 0.005) (Table 4). There were no between-group sex differences regarding medications taken during the night of the PSG (Table 2).

**Table 4.** Sex Differences in Subjective Quality of Life

	Men	Women
High Score Indicative of Better Health		
Ability to Participate	42 ± 1.7	39 ± 1.2
Cognition^***^	**45 ± 1.9**	**35 ± 2.7**
Communication^***^	**78 ± 3.5**	**44 ± 6.9**
Positive Affect & Well Being^*^	**53 ± 1.7**	**47 ± 1.8**
Satisfaction with Social Role/Activities^*^	**45 ± 1.2**	**40 ± 1.1**
Upper Extremity Motor Function^*^	**46 ± 1.7**	**36 ± 3.1**
Lower Extremity Motor Function^*^	**44 ± 2.1**	**35 ± 2.6**
Low Score Indicative of Better Health		
Depression^*^	**49 ± 1.6**	**54 ± 1.9**
Emotional/Behavioral Dyscontrol^*^	**47 ± 2.2**	**57 ± 3.6**
Fatigue^***^	**43 ± 1.9**	**54 ± 1.8**
Anxiety	52 ± 1.3	58 ± 2.8
Sleep Disturbance^***^	**47 ± 2.5**	**57 ± 2.1**
Stigma^*^	**51 ± 1.5**	**56 ± 1.8**

Neurological Quality of Life T scores are shown as mean ± standard error of the mean. Significance is demonstrated by ^*^p < 0.05, ^**^p < 0.005, ^***^p < 0.005.

### Sex differences in sleep macrostructure

**FIG. 1.**
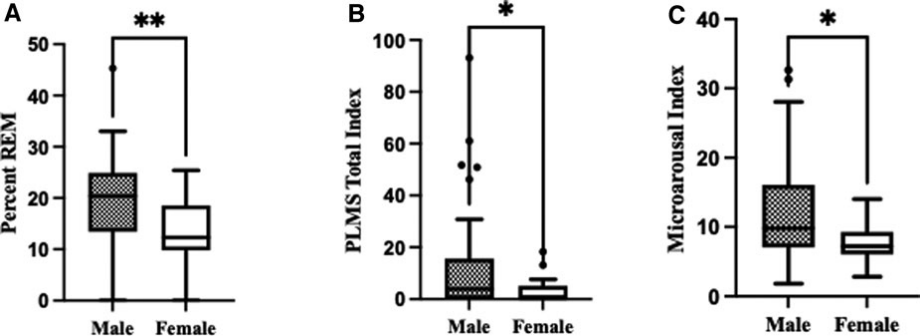
Sex differences in sleep macrostructure. (**A**) Men had higher percent time in the REM sleep stage, (**B**) more PLMS, and (**C**) more microarousals compared to women. Each value represents the mean ± standard error of the mean; *p < 0.05, **p < 0.005. PLMS, periodic limb movements during sleep; REM, rapid eye movement.

No sex effects were observed for total sleep time (338 min ±12 SEM), sleep latency (21 min ±4 SEM), wakefulness after sleep onset (WASO; 73 min ±4 SEM), and subjective sleepiness according to the Epworth Sleepiness Scale (6 min ±0.1 SEM). Overall, average percent time for sleep stages was: N1 (11 min ±1 SEM); N2 (60 min ±2 SEM); N3 (11 min ±2 SEM); and REM (18 min ±1 SEM). Analysis of sleep stages indicated a higher percent time in REM sleep for men (*p* < 0.005; Fig. 1A), whereas women showed a tendency to have a higher percent time in N2 (*p* = 0.08). Men had more periodic limb movements during sleep (PLMS; *p* < 0.05) and had a higher microarousal index per hour (*p* < 0.05; Fig. 1B,C). No significant sex differences were observed when analyzing apnea and hypopnea measures.

### Association between sleep stages and memory

**FIG. 2.**
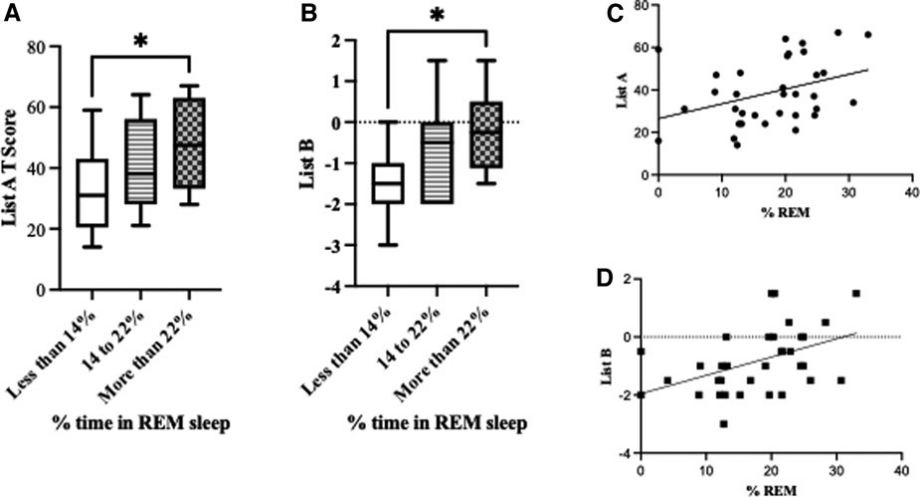
Time spent in REM was associated with performance in the California Verbal Learning Test. (**A**) Subjects with >22% REM sleep stage had better list A and (**B**) list B recall. Percent time in REM sleep stage was positively associated with better recall in (**C**) list A and (**D**) list B. Each value represents the mean ± standard error of the mean; *p < 0.05. REM, rapid eye movement.

A significant association between percent time in REM and immediate recall for CVLT-List A (trials 1–5) was observed. List A requires the subject to recall a list over five trials. Tukey's corrected comparison of scores below and above the normative percentage of REM sleep showed a better performance in CVLT-List A in those subjects with High-Range-REM compared to Low-Range-REM (*p* < 0.05). This was supported by a main effect for percentage of REM sleep in CVLT-List A (*F*_(2,31)_ = 3.4, *p* < 0.05; Fig. 2A). Simple linear regression revealed that the amount of REM accounted for 13% (*F*_(1,32)_ = 4.8, *p* < 0.05) of the variance in immediate recall of CVLT-List A (Fig. 2C).

Likewise, a significant association between percent time in REM and immediate recall for CVLT-List-B was found. List B is an interference list administered immediately after List A that requires the subject to recall after one trial. Tukey's corrected pairwise comparisons showed a better performance in the interference list recall (CVLT-List B) in those subjects with High-Range-REM compared to Low-Range-REM (*p* < 0.05). This was supported by a main effect for percent REM sleep in CVLT-List B (*F*_(2,31)_ = 4.1, *p* < 0.05; Fig. 2B). Simple linear regression revealed that the amount of REM accounted for 20% (*F*_(1,32)_ = 8.2, *p* < 0.05) of the variance of CVLT-List B (Fig. 2D).

**FIG. 3.**
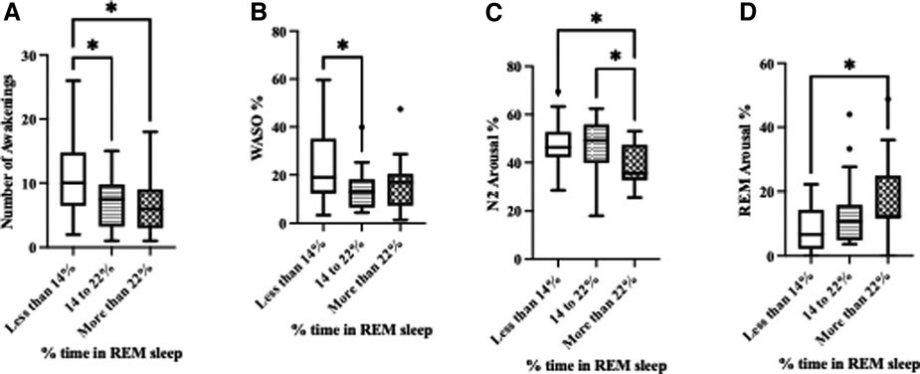
Subjects with <14% REM sleep showed a higher number of (**A**) awakenings, (**B)** percent WASO, and (**C**) percent arousals. (**D**) REM sleep arousals were higher in those subjects with >22% REM sleep stage. Each value represents the mean ± standard error of the mean; *p < 0.05. REM, rapid eye movement; WASO, wake after sleep onset.

Low-Range-REM was associated with a higher number of awakenings and WASO% (*p* < 0.05). This was supported by main effects for Awakenings (*F*_(2,54)_ = 5.9, *p* < 0.005) and WASO% (*F*_(2,54)_ = 3.5, *p* < 0.05; Fig. 3A,B). Pairwise comparisons also revealed that percent arousal during N2 was higher in those subjects with Low- and Mid-Range-REM compared to those with High-Range-REM (Fig. 3C). This was supported by a significant main effect (*F*_(2,53)_ = 4.6, *p* < 0.05). As expected, the percentage of arousals during REM sleep was higher in the High-Range-REM group (*p* < 0.05), with a significant main effect (*F*_(2,53)_ = 4.7, *p* < 0.05; Fig. 3D).

### Impact of medications on REM sleep

**FIG. 4.**
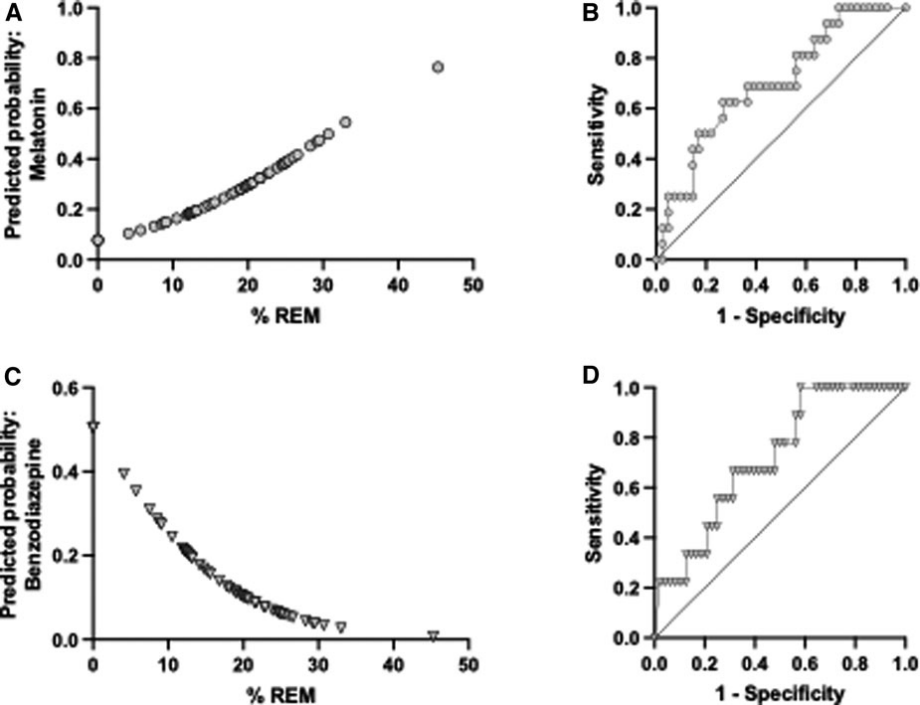
(**A**) Higher percent time in REM sleep was a significant predictor of melatonin utilization. (**B**) ROC curve for melatonin utilization versus time spent in REM sleep stage. (**C**) Lower percent time in REM sleep was a predictor of benzodiazepine utilization. (**D**) ROC curve for benzodiazepine utilization versus time spent in REM sleep stage. REM, rapid eye movement; ROC, receiver operating characteristic.

Results from logistic regression analysis suggest that the amount of REM sleep is a significant predictor of melatonin utilization, with an odds ratio of 1.08, indicating that for each unit increase in REM sleep, there is an associated increase in the odds of melatonin utilization (Fig. 4A). A significant Z value of 2.01 (*p* < 0.05) was observed, further demonstrating that the slope of the regression line was significant. Further, the likelihood ratio test resulted in a log-likelihood ratio (G squared) of 4.739 (*p* < 0.05), indicating a strong association between REM sleep and melatonin utilization. Additionally, the area under the ROC curve was 0.6997, with a 95% confidence interval of 0.55–0.85 (*p* < 0.05; Fig. 4B). These results suggest a statistically significant association between REM sleep and melatonin utilization.

In contrast, lower percent REM time was significantly associated with benzodiazepine use (z value = 2.2, *p* < 0.05). The odds ratio for this association was 0.8965, indicating a lower likelihood of benzodiazepine use among those with lower percent REM time (Fig. 4C). The log-likelihood ratio was 5.5 (*p* < 0.05). The area under the curve was 0.7 (95% confidence interval 0.56–0.88, *p* = 0.05; Fig. 4D). These results suggest that lower percent REM time is associated with lower likelihood of benzodiazepine use.

## Discussion

### Disruption in sleep architecture is sex dependent

Women reported more depression and poorer quality of life compared to men. Clinician-rated measures also showed that the women in this cohort were more impaired compared to men, particularly in the MPAI ability subscale that focuses on sensory, motor, and cognitive abilities.^43^ Depression and anxiety are more frequently found in women.^44,45^ These findings are in accordance with other studies reporting worse post-traumatic outcomes in women.^5,6^

Sex differences in neurobiological processes underlying depression and anxiety are likely to impact neuronal networks during sleep.^44,46,47^ Here, we observed that men spent significantly more time in REM sleep, whereas women spent a significant portion of total sleep time in N2. In contrast with these findings, other PSG studies indicate that healthy non-injured women show more REM sleep duration, as well as better sleep efficiency and longer periods of slow-wave sleep.^29,39,48^ Thus, in the general population, women seem to have better objective sleep, but more self-reported insomnia compared to men.^28^ Comorbidities observed after TBI may be associated with REM alterations.^49^ Results from this female cohort reveal more depression and decreased REM sleep. These results contrast with other studies suggesting that increased REM can be a biomarker for depression.^50,51^ Nevertheless, more recent studies have demonstrated that the link between REM disinhibition and depression is not reliable.^52^

Reports of REM sleep post-TBI are mixed with some studies showing decreases in REM sleep,^53–55^ yet systematic reviews and meta-analyses report a trend for either decreases in REM or no changes in REM post-TBI.^56,57^ However, to our knowledge, sex was not analyzed as a primary variable in any of these studies. In addition to the potential variability attributed to sex, discrepancies in PSG findings are not surprising given the heterogeneity of TBI, use of medications, and differences in equipment utilized. Pre-clinical studies have found that experimental TBI predominantly increases NREM sleep^58–61^ and has been found to interfere with enriched environment induced increases in REM sleep.^62^ It should be noted that sleep stages in murine models of TBI are usually presented as REM and NREM, with NREM being categorized as a single stage.^22^ Sex-dependent effects, such as persistent hypersomnia in females, have also been shown after experimental TBI.^63,64^

### Association between REM sleep and short-term episodic memory

Whereas human and animal studies have shown an increased prevalence of post-traumatic SWDs, the extent to which these disturbances impact chronic outcome remains unclear. Here, we found an association between percent time in REM and better recall scores. Others have observed an association between amount of REM and memory potential.^65^ It is widely accepted that both N3 and REM play a critical role in learning memory.^16,22,66,67^ N3 is characterized by low-frequency, high-amplitude waves and appears to be strongly implicated in cognitive processing. Interestingly, an association between N3 and cognitive function was not observed. It should be noted that the average time spent in N3 was lower than the normative value across all TBI subjects.^38,40,41,68^

### Impact of medications on REM sleep

TBI patients are frequently on several medications that may impact sleep architecture. Most of the study subjects were on antidepressant therapy, of which the majority were taking a selective serotonin reuptake inhibitor (SSRI; 45%), which are known to suppress REM. The duration of treatment was unknown. Despite this, no effects on REM sleep were observed. Effects on REM are maximal early in treatment and diminish significantly over time.^32^

Melatonin (nacetyl-5-methoxytryptamine) is widely utilized as an insomnia treatment.^33^ Accordingly, melatonin use was registered in 28% of the subjects and was associated with increases in REM sleep duration. The effect of melatonin administration on sleep architecture is equivocal.^69^ In accordance with the present described results, others have reported melatonin-associated increases in REM sleep.^70,71^ Correspondingly, low endogenous melatonin is accompanied by REM sleep reductions.^72^ However, other studies show no increases in REM subsequent to melatonin administration.^73,74^ Effects of melatonin administration are complex because of extrinsic variables, such as time of administration and duration of treatment, as well as intrinsic variables, such as differential and dynamic properties of melatonin receptors.^75,76^ This is further complicated by the impact that TBI has on melatonin receptors.^77^ Clearly, more investigation on the subject is necessary.

Use of benzodiazepines was reported in 16% of the study cohort and was associated with significantly reduced percent time in REM sleep. Benzodiazepine use has also been associated with REM and slow wave sleep reductions in healthy volunteers.^78,79^

### Limitations

Results from this study were based on clinical recordings from single-night PSGs, bringing the potential impact of first night effect (FNE) on the data collected. Considering the potential impact of FNE on REM sleep,^80^ it is possible that this effect contributed to the sex differences observed in REM. However, this explanation is made less likely when we consider that there were no observed sex differences in total sleep time.

Whereas we can contemplate the possibility that decreases in REM sleep contributed to the cognitive performance in this cohort of women, because of logistical reasons, we did not have the statistical power to make conclusive claims regarding the impact of REM on verbal memory utilizing sex as a biological variable. Likewise, we did not have the statistical power to determine the impact of medications on REM duration. However, it seems unlikely that elevated REM sleep in men was attributable to medication use, given that no sex effects were observed for melatonin or benzodiazepine utilization. In addition, menstrual cycle stage was not recorded at the time of the PSG. Considering that REM is impacted by changes in the menstrual cycle,^81,82^ it is possible that the observed sex differences are, at least in part, attributable to menstrual cycle stage.

## Conclusion

Findings from this observational study support the hypothesis that decreases in REM sleep may contribute to chronic disability. Results also indicate a sex-dependent decrease in REM sleep that may contribute to disparities in post-TBI outcome. It is becoming increasingly evident that sex provides insight on sleep pathophysiology after injury and more research is necessary, given the notable scarcity of sleep studies focusing on sex differences post-TBI. A better understanding of how changes in sleep impact outcome potential will be useful in defining targeted interventions.

## Abbreviations Used

BDI: Beck Depression Inventory II
CVLT: California Verbal Learning Test 2
FNE: first night effect
GCS: Glasgow Coma Scale
MPAI: Mayo Portland Adaptability Inventory 4
Neuro QoL: Neurological Quality of Life Questionnaire
NREM: non-rapid eye movement
PLMS: periodic limb movements during sleep
PSG: polysomnography
REM: rapid eye movement
ROC: receiver operating characteristic
SEM: standard error of the mean
SSRI: selective serotonin reuptake inhibitor
SWDs: sleep-wake disturbances
TBI: traumatic brain injury
WASO: wakefulness after sleep onset

## References

[1] McAllister TW, Flashman LA, McDonald BC, et al. Mechanisms of working memory dysfunction after mild and moderate TBI: evidence from functional MRI and neurogenetics. J Neurotrauma 2006;23(10):1450–1467; doi: 10.1089/neu.2006.23.1450 17020482

[2] Levin HS. Memory deficit after closed head injury. J Clin Exp Neuropsychol 1990;12(1):129–153; doi: 10.1080/01688639008400960 2406280

[3] Stocchetti N, Zanier ER. Chronic impact of traumatic brain injury on outcome and quality of life: a narrative review. Crit Care 2016;20(1):148; doi: 10.1186/s13054-016-1318-1 27323708 PMC4915181

[4] Farace E, Alves WM. Do women fare worse: a metaanalysis of gender differences in traumatic brain injury outcome. J Neurosurg 2000;93(4):539–545; doi: 10.3171/jns.2000.93.4.0539 11014529

[5] Gupte R, Brooks W, Vukas R, et al. Sex differences in traumatic brain injury: what we know and what we should know. J Neurotrauma 2019;36(22):3063–3091; doi: 10.1089/neu.2018.6171 30794028 PMC6818488

[6] Mikolić A, van Klaveren D, Groeniger JO, et al.; CENTER-TBI Participants and Investigators. Differences between men and women in treatment and outcome after traumatic brain injury. J Neurotrauma 2021;38(2):235–251; doi: 10.1089/neu.2020.7228 32838645

[7] Lequerica AH, Weber E, Dijkers MP, et al. Factors associated with the remission of insomnia after traumatic brain injury: a traumatic brain injury model systems study. Brain Inj 2020;34(2):187–194; doi: 10.1080/02699052.2019.1682193 31640430 PMC9014829

[8] Fogelberg DJ, Hoffman JM, Dikmen S, et al. Association of sleep and co-occurring psychological conditions at 1 year after traumatic brain injury. Arch Phys Med Rehabil 2012;93(8):1313–1318; doi: 10.1016/j.apmr.2012.04.031 22840828

[9] Griesbach GS, Masel BE, Helvie RE, et al. The impact of traumatic brain injury on later life: effects on normal aging and neurodegenerative diseases. J Neurotrauma 2018;35(1):17–24; doi: 10.1089/neu.2017.5103 28920532

[10] Sandsmark DK, Elliott JE, Lim MM. Sleep-wake disturbances after traumatic brain injury: synthesis of human and animal studies. Sleep 2017;40(5):zsx044; doi: 10.1093/sleep/zsx044 28329120 PMC6251652

[11] Fulda S, Schulz H. Cognitive dysfunction in sleep disorders. Sleep Med Rev 2001;5(6):423–445; doi: 10.1053/smrv.2001.0157 12531152

[12] Motivala SJ. Sleep and inflammation: psychoneuroimmunology in the context of cardiovascular disease. Ann Behav Med 2011;42(2):141–152; doi: 10.1007/s12160-011-9280-2 21604067

[13] Lange T, Dimitrov S, Born J. Effects of sleep and circadian rhythm on the human immune system. Ann N Y Acad Sci 2010;1193(1):48–59; doi: 10.1111/j.1749-6632.2009.05300.x 20398008

[14] Cable J, Schernhammer E, Hanlon EC, et al. Sleep and circadian rhythms: pillars of health—a Keystone Symposia report. Ann N Y Acad Sci 2021;1506(1):18–34; doi: 10.1111/nyas.14661 34341993 PMC8688158

[15] Dams-O'Connor K, Spielman L, Singh A, et al.; TRACK-TBI Investigators. The impact of previous traumatic brain injury on health and functioning: a TRACK-TBI study. J Neurotrauma 2013;30:2014–2020; doi: 10.1089/neu.2013.3049 23924069 PMC3868372

[16] MacDonald KJ, Cote KA. Contributions of post-learning REM and NREM sleep to memory retrieval. Sleep Med Rev 2021;59:101453; doi: 10.1016/j.smrv.2021.101453 33588273

[17] Feld GB, Born J. Sculpting memory during sleep: concurrent consolidation and forgetting. Curr Opin Neurobiol 2017;44:20–27; doi: 10.1016/j.conb.2017.02.012 28278432

[18] Fogel SM, Smith CT. The function of the sleep spindle: a physiological index of intelligence and a mechanism for sleep-dependent memory consolidation. Neurosci Biobehav Rev 2011;35(5):1154–1165; doi: 10.1016/j.neubiorev.2010.12.003 21167865

[19] Avital A, Richter-Levin G. Exposure to juvenile stress exacerbates the behavioural consequences of exposure to stress in the adult rat. Int J Neuropsychopharmacol 2005;8(2):163–173; doi: 10.1017/S1461145704004808 15546500

[20] Grosmark AD, Mizuseki K, Pastalkova E, et al. REM sleep reorganizes hippocampal excitability. Neuron 2012;75(6):1001–1007; doi: 10.1016/j.neuron.2012.08.015 22998869 PMC3608095

[21] Kuhn M, Wolf E, Maier JG, et al. Sleep recalibrates homeostatic and associative synaptic plasticity in the human cortex. Nat Commun 2016;7:12455; doi: 10.1038/ncomms12455 27551934 PMC4996971

[22] Diekelmann S, Born J. The memory function of sleep. Nat Rev Neurosci 2010;11(2):114–126; doi: 10.1038/nrn2762 20046194

[23] Sonni A, Spencer RMC. Sleep protects memories from interference in older adults. Neurobiol Aging 2015;36(7):2272–2281; doi: 10.1016/j.neurobiolaging.2015.03.010 25890819 PMC4457602

[24] Suh S, Cho N, Zhang J. Sex differences in insomnia: from epidemiology and etiology to intervention. Curr Psychiatry Rep 2018;20(9):69; doi: 10.1007/s11920-018-0940-9 30094679

[25] Morin CM, Jarrin DC. Epidemiology of insomnia: prevalence, course, risk factors, and public health burden. Sleep Med Clin 2022;17(2):173–191; doi: 10.1016/j.jsmc.2022.03.003 35659072

[26] Van Eycken S, Neu D, Newell J, et al. Sex-related differences in sleep-related PSG parameters and daytime complaints in a clinical population. Nat Sci Sleep 2020;12:161–171; doi: 10.2147/NSS.S235642 32110127 PMC7037102

[27] Eliasson AH, Kashani MD, Howard RS, et al.; Integrative Cardiac Health Project Registry. Fatigued on Venus, sleepy on Mars—gender and racial differences in symptoms of sleep apnea. Sleep Breath 2015;19(1):99–107; doi: 10.1007/s11325-014-0968-y 24633816

[28] Wickwire EM, Albrecht JS, Capaldi VF II, et al.; Transforming Research and Clinical Knowledge in Traumatic Brain Injury (TRACK-TBI) Investigators. Trajectories of insomnia in adults after traumatic brain injury. JAMA Netw Open 2022;5(1):e2145310; doi: 10.1001/jamanetworkopen.2021.45310 35080600 PMC8792888

[29] Goel N, Kim H, Lao RP. Gender differences in polysomnographic sleep in young healthy sleepers. Chronobiol Int 2005;22(5):905–915; doi: 10.1080/07420520500263235 16298775

[30] Busto R, Dietrich WD, Globus MY, et al. Small differences in intraischemic brain temperature critically determine the extent of ischemic neuronal injury. J Cereb Blood Flow Metab 1987;7(6):729–738; doi: 10.1038/jcbfm.1987.127 3693428

[31] Wichniak A, Wierzbicka A, Walęcka M, Jernajczyk W. Effects of antidepressants on sleep. Curr Psychiatry Rep 2017;19(9):63; doi: 10.1007/s11920-017-0816-4 28791566 PMC5548844

[32] Wilson S, Argyropoulos S. Antidepressants and sleep: a qualitative review of the literature. Drugs 2005;65(7):927–947; doi: 10.2165/00003495-200565070-00003 15892588

[33] Dujardin S, Pijpers A, Pevernagie D. Prescription drugs used in insomnia. Sleep Med Clin 2020;15(2):133–145; doi: 10.1016/j.jsmc.2020.02.002 32386689

[34] De Crescenzo F, D'Alò GL, Ostinelli EG, et al. Comparative effects of pharmacological interventions for the acute and long-term management of insomnia disorder in adults: a systematic review and network meta-analysis. Lancet 2022;400(10347):170–184; doi: 10.1016/S0140-6736(22)00878-9 35843245

[35] Ozdemir PG, Karadag AS, Selvi Y, et al. Assessment of the effects of antihistamine drugs on mood, sleep quality, sleepiness, and dream anxiety. Int J Psychiatry Clin Pract 2014;18(3):161–168. doi: 10.3109/13651501.2014.907919 24673474

[36] Liguori C, Toledo M, Kothare S. Effects of anti-seizure medications on sleep architecture and daytime sleepiness in patients with epilepsy: a literature review. Sleep Med Rev 2021;60:101559; doi: 10.1016/j.smrv.2021.101559 34710770

[37] Carnovale C, Perrotta C, Baldelli S, et al. Antihypertensive drugs and brain function: mechanisms underlying therapeutically beneficial and harmful neuropsychiatric effects. Cardiovasc Res 2023;119(3):647–667; doi: 10.1093/cvr/cvac110 35895876 PMC10153433

[38] Mitterling T, Högl B, Schönwald SV, et al. Sleep and respiration in 100 healthy Caucasian sleepers—a polysomnographic study according to American Academy of Sleep Medicine standards. Sleep 2015;38(6):867–875; doi: 10.5665/sleep.4730 25515109 PMC4434553

[39] Luca G, Haba Rubio J, Andries D, et al. Age and gender variations of sleep in subjects without sleep disorders. Ann Med 2015;47(6):482–491; doi: 10.3109/07853890.2015.1074271 26224201

[40] Carskadon MA, Dement WC. Normal Human Sleep: An Overview. In: Principles and Practice of Sleep Medicine. 5th ed. (Kryger MH, Roth T, Dement WC. eds.) Elsevier: St Louis, MO; 2011; pp. 16–26.

[41] Della Monica C, Johnsen S, Atzori G, et al. Rapid eye movement sleep, sleep continuity and slow wave sleep as predictors of cognition, mood, and subjective sleep quality in healthy men and women, aged 20–84 years. Front Psychiatry 2018;9:255; doi: 10.3389/fpsyt.2018.00255 29988413 PMC6024010

[42] Taylor CA, Bell JM, Breiding MJ, et al. Traumatic brain injury-related emergency department visits, hospitalizations, and deaths—United States, 2007 and 2013. MMWR Surveill Summ. 2017;66(9):1–16; doi: 10.15585/mmwr.ss6609a1 PMC5829835 28301451

[43] Bellon K, Malec JF, Kolakowsky-Hayner SA. Mayo-Portland Adaptability Inventory-4. J Head Trauma Rehabil 2012;27(4):314–316; doi: 10.1097/HTR.0b013e3182562f04 22767075

[44] Eid RS, Gobinath AR, Galea LAM. Sex differences in depression: insights from clinical and preclinical studies. Prog Neurobiol 2019;176:86–102; doi: 10.1016/j.pneurobio.2019.01.006 30721749

[45] Altemus M, Sarvaiya N, Neill Epperson C. Sex differences in anxiety and depression clinical perspectives. Front Neuroendocrinol 2014;35(3):320–330; doi: 10.1016/j.yfrne.2014.05.004 24887405 PMC4890708

[46] Bangasser DA, Cuarenta A. Sex differences in anxiety and depression: circuits and mechanisms. Nat Rev Neurosci 2021;22(11):674–684; doi: 10.1038/s41583-021-00513-0 34545241

[47] Hein M, Lanquart JP, Loas G, et al. Alterations of neural network organization during REM sleep in women: implication for sex differences in vulnerability to mood disorders. Biol Sex Differ 2020;11(1):22; doi: 10.1186/s13293-020-00297-5 32334638 PMC7183628

[48] Kobayashi R, Kohsaka M, Fukuda N, et al. Gender differences in the sleep of middle-aged individuals. Psychiatry Clin Neurosci 1998;52(2):186–187; doi: 10.1111/j.1440-1819.1998.tb01021.x 9628142

[49] Cowdin N, Kobayashi I, Mellman TA. Theta frequency activity during rapid eye movement (REM) sleep is greater in people with resilience versus PTSD. Exp Brain Res 2014;232(5):1479–1485; doi: 10.1007/s00221-014-3857-5 24531640 PMC4449337

[50] Steiger A, Pawlowski M. Depression and sleep. Int J Mol Sci 2019;20(3):607; doi: 10.3390/ijms20030607 30708948 PMC6386825

[51] Strawbridge R, Young AH, Cleare AJ. Biomarkers for depression: recent insights, current challenges and future prospects. Focus (Am Psychiatr Publ) 2018;16(2):194–209; doi: 10.1176/appi.focus.16206 32015707 PMC6526845

[52] Riemann D, Krone LB, Wulff K, et al. Sleep, insomnia, and depression. Neuropsychopharmacology 2020;45(1):74–89; doi: 10.1038/s41386-019-0411-y 31071719 PMC6879516

[53] Schreiber S, Barkai G, Gur-Hartman T, et al. Long-lasting sleep patterns of adult patients with minor traumatic brain injury (mTBI) and non-mTBI subjects. Sleep Med. 2008;9(5):481–7. 17638592 10.1016/j.sleep.2007.04.014

[54] Parcell DL, Ponsford JL, Redman JR, et al. Poor sleep quality and changes in objectively recorded sleep after traumatic brain injury: a preliminary study. Arch Phys Med Rehabil 2008;89(5):843–850; doi: 10.1016/j.apmr.2007.09.057 18452730

[55] Mollayeva T, Colantonio A, Cassidy JD, et al. Sleep stage distribution in persons with mild traumatic brain injury: a polysomnographic study according to American Academy of Sleep Medicine standards. Sleep Med 2017;34:179–192; doi: 10.1016/j.sleep.2017.02.021 28522089

[56] Mantua J, Grillakis A, Mahfouz SH, et al. A systematic review and meta-analysis of sleep architecture and chronic traumatic brain injury. Sleep Med Rev 2018;41:61–77; doi: 10.1016/j.smrv.2018.01.004 29452727

[57] Grima N, Ponsford J, Rajaratnam SM, et al. Sleep disturbances in traumatic brain injury: a meta-analysis. J Clin Sleep Med 2016;12(3):419–428; doi: 10.5664/jcsm.5598 26564384 PMC4773614

[58] Lim MM, Elkind J, Xiong G, et al. Dietary therapy mitigates persistent wake deficits caused by mild traumatic brain injury. Sci Transl Med 2013;5(215):215ra173. 10.1126/scitranslmed.3007092 PMC3951738 24337480

[59] Konduru SS, Wallace EP, Pfammatter JA, et al. Sleep-wake characteristics in a mouse model of severe traumatic brain injury: relation to posttraumatic epilepsy. Epilepsia Open 2021;6(1):181–194; doi: 10.1002/epi4.12462 33681661 PMC7918302

[60] Noain D, Büchele F, Schreglmann SR, et al. Increased sleep need and reduction of tuberomammillary histamine neurons after rodent traumatic brain injury. J Neurotrauma 2018;35(1):85–93; doi: 10.1089/neu.2017.5067 28762870

[61] Thomasy HE, Opp MR. Hypocretin mediates sleep and wake disturbances in a mouse model of traumatic brain injury. J Neurotrauma 2019;36(5):802–814; doi: 10.1089/neu.2018.5810 30136622 PMC6387567

[62] Borniger JC, Ungerleider K, Zhang N, et al. Repetitive brain injury of juvenile mice impairs environmental enrichment-induced modulation of REM sleep in adulthood. Neuroscience 2018;375:74–83; doi: 10.1016/j.neuroscience.2018.01.064 29432885 PMC6668616

[63] Portillo E, Zi X, Kim Y, et al. Persistent hypersomnia following repetitive mild experimental traumatic brain injury: roles of chronic stress and sex differences. J Neurosci Res 2023;101(6):843–865; doi: 10.1002/jnr.25165 36624699

[64] Rowe RK, Harrison JL, O'Hara BF, et al. Diffuse brain injury does not affect chronic sleep patterns in the mouse. Brain Inj 2014;28(4):504–510; doi: 10.3109/02699052.2014.888768 24702469 PMC7482552

[65] Lafortune M, Gagnon JF, Martin N, et al. Sleep spindles and rapid eye movement sleep as predictors of next morning cognitive performance in healthy middle-aged and older participants. J Sleep Res 2014;23(2):159–167; doi: 10.1111/jsr.12108 24245769

[66] Li W, Ma L, Yang G, et al. REM sleep selectively prunes and maintains new synapses in development and learning. Nat Neurosci 2017;20(3):427–437; doi: 10.1038/nn.4479 28092659 PMC5535798

[67] Feld GB, Diekelmann S. Sleep smart-optimizing sleep for declarative learning and memory. Front Psychol 2015;6:622; doi: 10.3389/fpsyg.2015.00622 26029150 PMC4428077

[68] Agha A, Rogers B, Mylotte D, et al. Neuroendocrine dysfunction in the acute phase of traumatic brain injury. Clin Endocrinol (Oxf) 2004;60(5):584–591; doi: 10.1111/j.1365-2265.2004.02023.x 15104561

[69] Ferracioli-Oda E, Qawasmi A, Bloch MH. Meta-analysis: melatonin for the treatment of primary sleep disorders. PLoS One 2013;8(5):e63773; doi: 10.1371/journal.pone.0063773 23691095 PMC3656905

[70] Kunz D, Mahlberg R, Müller C, et al. Melatonin in patients with reduced REM sleep duration: two randomized controlled trials. J Clin Endocrinol Metab 2004;89(1):128–134; doi: 10.1210/jc.2002-021057 14715839

[71] Cajochen C, Kräuchi K, Möri D, et al. Melatonin and S-20098 increase REM sleep and wake-up propensity without modifying NREM sleep homeostasis. Am J Physiol 1997;272(4 Pt 2):R1189–R1196. 9140019 10.1152/ajpregu.1997.272.4.R1189

[72] Mahlberg R, Kienast T, Hädel S, et al. Degree of pineal calcification (DOC) is associated with polysomnographic sleep measures in primary insomnia patients. Sleep Med 2009;10(4):439–445; doi: 10.1016/j.sleep.2008.05.003 18755628

[73] Xu H, Zhang C, Qian Y, et al. Efficacy of melatonin for sleep disturbance in middle-aged primary insomnia: a double-blind, randomised clinical trial. Sleep Med 2020;76:113–119; doi: 10.1016/j.sleep.2020.10.018 33157425

[74] Stone BM, Turner C, Mills SL, et al. Hypnotic activity of melatonin. Sleep 2000;23(5):663–669. 10947034

[75] Gobbi G, Comai S. Differential function of melatonin MT_1_ and MT_2_ receptors in REM and NREM sleep. Front Endocrinol 2019;10:87; doi: 10.3389/fendo.2019.00087 PMC6407453 30881340

[76] Zhdanova IV. Melatonin as a hypnotic: pro. Sleep Med Rev 2005;9(1):51–65; doi: 10.1016/j.smrv.2004.04.003 15649738

[77] Osier ND, Pham L, Pugh BJ, et al. Brain injury results in lower levels of melatonin receptors subtypes MT1 and MT2. Neurosci Lett 2017;650:18–24; doi: 10.1016/j.neulet.2017.03.053 28377323 PMC5886725

[78] Kilduff T, Mendelson WB. Hypnotic Medications: Mechanisms of Action and Pharmacologic Effects. In: Principles and Practice of Sleep Medicine (Sixth Edition). (Kryger M, Roth T, Dement WC. eds.) Elsevier: Philadelphia, PA; 2017.

[79] Roth T. Insomnia: definition, prevalence, etiology, and consequences. J Clin Sleep Med 2007;3(5 Suppl):S7–S10. 17824495 PMC1978319

[80] Byun JH, Kim KT, Moon HJ, et al. The first night effect during polysomnography, and patients' estimates of sleep quality. Psychiatry Res 2019;274:27–29; doi: 10.1016/j.psychres.2019.02.011 30776709

[81] Dorsey A, de Lecea L, Jennings KJ. Neurobiological and hormonal mechanisms regulating women's sleep. Front Neurosci 2020;14:625397; doi: 10.3389/fnins.2020.625397 33519372 PMC7840832

[82] Morssinkhof MWL, van Wylick DW, Priester-Vink S, et al. Associations between sex hormones, sleep problems and depression: a systematic review. Neurosci Biobehav Rev 2020;118:669–680; doi: 10.1016/j.neubiorev.2020.08.006 32882313

